# SENIOR CITIZEN SCIENCE FOR AGE-FRIENDLY COMMUNITIES

**DOI:** 10.1093/geroni/igad104.1041

**Published:** 2023-12-21

**Authors:** Helen Barrie

**Affiliations:** University of South Australia, Adelaide, South Australia, Australia

## Abstract

This presentation explores the use of citizen science with older participants; highlighting the potential for this underutilized methodology to create better, data driven, outcomes for age friendly cities and communities. Local neighborhoods have major impacts on older people’s mobility, autonomy and independence, and quality of life. The WHO has developed clear guidelines for achieving age-friendly cities or communities. Design and delivery of quality public spaces that meet the age friendly guidelines should promote health and well-being, social engagement with others, and engagement with the environment but little data is currently collected about how older people perceive or use public spaces. Daily experiences, often over long periods of time, mean older residents have acquired intimate first-hand knowledge of their neighborhoods, and thus, may be more qualified than experts to assess the age friendly qualities of neighborhood public spaces. A citizen science approach addresses this need for a deeper understanding of how public spaces are used and viewed by older residents. Through multiple citizen science projects over the past five years, we have co-designed and trialed a ‘senior’ citizen science approach to auditing and evaluating the age friendliness of public spaces. Our research shows that citizen science allows for richer, deeper qualitative, quantitative and longitudinal data that incorporates lived experience perspectives; produces better project designs and outcomes; develops a cohort of older people interested and engaged in research, and engages and trains people to apply a critical ‘age friendly’ perspective to their neighborhoods and communities enabling them to be future change agents.

